# Biology Open 2025 – a year in review

**DOI:** 10.1242/bio.062560

**Published:** 2026-03-30

**Authors:** Daniel Gorelick

**Affiliations:** Editor-in-Chief, Biology Open

This is my favourite time of year because it is the time when we at Biology Open (BiO) exercise maximum transparency. We provide the public with detailed bibliometric data on how BiO performed during the previous calendar year. Let the sunlight of transparency shine during the darkest days of the year in the Northern Hemisphere!

2025 was an especially exciting year for us. In January, we introduced transparent peer review, and, from March onwards, we phased out conventional peer review. Since then, Fast & Fair peer review has been the default pathway for all new submissions. We provide authors a decision, with review, within 7 working days of submission. We can do this because we precontract with and pay peer reviewers (220 GBP/manuscript). We hold reviewers accountable. If they don't submit a quality review on time, then they don't get paid.

We're scientists, so of course there are caveats. Our goal was for every new submission to go through Fast & Fair peer review; but, in a few cases, we lacked precontracted reviewers with relevant expertise to review a manuscript, so that manuscript went through conventional peer review. Manuscripts that were reviewed at one of our sister journals, rejected and then transferred to BiO also did not go through Fast & Fair peer review. None of our sister journals precontract with or pay reviewers, so if we wanted to send a transferred manuscript back to the original reviewers, we could not do so through Fast & Fair because the original reviewers were not under contract. In this Editorial, I'll focus on overall statistics. For a specific analysis of Fast & Fair peer review, see an earlier preprint (Gorelick and Clark, 2025 preprint).

## How would I assess our performance in 2025?

Submissions increased by 15%, with an 18% increase in the number of published, peer-reviewed manuscripts in 2025 versus 2024 (Table 1). The median time from submission to first decision with reviews was 8 days, a decline of 80% compared to 2024, when most manuscripts went through conventional peer review (Fast & Fair was a limited experiment tested on only ∼20 manuscripts in July to December 2024). The median time from submission to final decision was 36 days, a decline of 45%. Keep in mind that once we provide authors an initial decision of revise and resubmit, the authors have up to 90 days to complete their revisions, and the amount of time the authors take is out of our control.

**Table 1.** BiO publication statistics for Research Articles and Methods & Techniques articles for 2023-2025

Research manuscript statistics (excludes reviews)	2023	2024	2025
Submissions (total, including transfers)	308	365	419
Published articles	100	129	152
Overall acceptance rate	35%	33%	35%
Editorial rejections	142	136	154
Submissions sent for peer review	144	208	189
Direct submissions	174	242	241
Transferred submissions	134	124	178
Transfers without reviews	81	99	130
Transfers with reviews	53	25	48
Transferred articles accepted without additional peer review	22	17	29
Transferred articles accepted (total)	51	58	68
Peer review statistics (days)			
Submission to final decision* (mean±s.d.)	70±54	77±60	55±52
Submission to final decision* (median)	59	65	36
Submission to editorial rejection (without peer review) (mean)	10±11	10±23	5±6
Submission to editorial rejection (without peer review) (median)	7	3	2
Submission to first decision following peer review (mean)	34±26	46±41	17±21
Submission to first decision following peer review (median)	33	39	8

*Includes decisions on accepted and rejected papers.

What makes this performance most astounding is that our overall acceptance rate stayed consistent at 33-35% during 2023, 2024 and 2025. This demonstrates that we did not compromise on the rigour of peer review despite publishing more manuscripts and providing authors with a faster turnaround time.

I would love to conclude that Fast & Fair caused the increase in submissions. Maybe some other change, like the introduction of a consistent and public rubric for how we evaluate manuscripts, or the fact that we publish the reviews of accepted manuscripts, was the driving factor behind the increase in submissions. Maybe this increase represents a general recovery from the reduced submissions during 2020-2022 (COVID-19 years), because submissions in 2024 were also up 19% compared to those in 2023. Time will tell. Our publisher authorised us to continue Fast & Fair for calendar year 2026, so I look forward to analysing those results next year.

None of this would be possible without the dedication and hard work of our staff, academic editors and peer reviewers, who have made 2025 a blockbuster year for BiO. Special thanks to all our peer reviewers, who are listed in the supplementary information. Thank you all for your dedication to BiO and The Company of Biologists.

Around the world, confidence in scientific publishing is being tested. Researchers are navigating funding instability, rising publication costs, paper mills, AI-generated manuscripts and a growing sense that the traditional systems meant to evaluate science are strained. Reforming peer review may seem incremental against challenges of that scale, but systemic change rarely happens all at once. It happens because people decide that one part of the system can work better. Fast & Fair is our contribution to that effort: a practical, tested model showing that speed, rigour and accountability are not mutually exclusive. If Fast & Fair can improve peer review at BiO, then similar principles can take root elsewhere.

## Supplementary Material

10.1242/biolopen.062560_sup1 Supplementary information
