# Supplementary material for: Biology Open 2025 – a year in review
Source: Biol Open. 2026 Mar 30;15(3):bio062560. doi: 10.1242/bio.062560 (PMC13072076; doi:10.1242/bio.062560)
Supplement: Supplementary information [file biolopen-15-062560-s1.pdf]

Reviewers for Biology Open, 2025

Fernando Abdulkader, University of São Paulo, Brazil

Hermann Aberle, Heinrich Heine University Düsseldorf, Germany

Olawale Adeyinka, The University of Alabama in Huntsville, USA

Priti Agarwal, National Centre for Biological Sciences, India

Natsumi Ageta-Ishihara, Toho University, Japan

Fernan Aguero, Universidad Nacional de San Martin (UNSAM), Argentina

Mohammad Ahmadianpour, National Institute for Genetic Engineering and Biotechnology, Islamic Republic of Iran

Giuseppe Aiello, University of Lausanne, Switzerland

Alper Akay, University of East Anglia, UK

Anna Akhmanove, Utrecht University, Netherlands

Leonardo Almeida-Souza, University of Helsinki, Finland

Linda Alrayes, Western University, Canada

A. Alwin Prem Anand, Eberhard Karls University of Tübingen, Germany

Mariana Angoa-Perez, Wayne State University, USA

Sofia Araujo, University of Barcelona, Spain

Krishan Ariyasiri, Geisel School of Medicine at Dartmouth, USA

Graham Askew, University of Leeds, UK

Domenico Azarnia Tehran, University of Padua, Italy

Scott Barbee, University of Denver, USA

Gonzalo Barceló, Adolfo Ibáñez University, Chile

El-Sayed Baz, KU Leuven, Belgium

Camilla Bean, Saint Camillus International University of Health and Medical Sciences, Italy

William Beckmann, Cambridge Stem Cell Institute, UK

Tobias Beigl, Ascendis Pharma, Germany

John Belcher, University of Minnesota, USA

Alexandre Benedetto, Lancaster University, UK

Anahi Binagui-Casas, University of Edinburgh, UK

Mélanie Bonhivers, University of Bordeaux, France

Kenny Breuer, Brown University, USA

Ariel Camp, University of Liverpool, UK

Tim Caro, University of Bristol, UK

Pierre Caron, Institut de biologie structurale (IBS), France

Jason Casler, Northwestern University, USA

Tamara Caspary, Emory University, USA

Fabrice Caudron, Institute of Molecular Genetics of Montpellier, France

Craig Ceol, University of Massachusetts Medical School, USA

Kara Cerveny, Reed College, USA

Joy Chakraborty, CSIR - Indian Institute of Chemical Biology, India

James Charles, University of Liverpool, UK

Marios Chatzigeorgiou, University of Bergen, Norway

Hui Chen, University of South Carolina, USA

Khong-Sam Chia, John Innes Centre, UK

Yueh Cho, Academia Sinica, Taiwan

Ifeoma Chukwuma, University of Nigeria, Nigeria

Osvaldo Contreras, Victor Chang Cardiac Research Institute, Australia

David Costantini, Tuscia University, Italy

James Crall, University of Wisconsin-Madison, USA

Enikö Csata, Museum and Institute of Zoology, Polish Academy of Sciences, Poland

Giovanni Dalmasso, Institut Químic de Sarrià, Ramon Llull University, Spain

Gabrielle Davidson, University of East Anglia, UK

Randall Davis, Texas A&M University at Galveston, USA

Jose de Celis, Autonomous University of Madrid, Spain

Peter Dearden, University of Otago, New Zealand

Sushanta Deb, Washington State University, USA

Paul Delgado-Olguin, SickKids Research Institute, Canada

Robert Dilley, Massachusetts General Hospital, USA

Eimear Dolan, University of São Paulo, Brazil

Paolo Domenici, Institute of Biophysics, Italy

Jacob Dunn, Anglia Ruskin University, UK

Kyle Elliott, McGill University, Canada

Nadja Engel, Rostock University Medical Center, Germany

Timothy Erickson, University of New Brunswick, Canada

Martin Estermann, National Institute of Environmental Health Sciences, USA

John Ewer, Valparaiso University, Chile

Andreas Fahlman, Fundación Oceanogràfic, Spain

Marie-Anne Felix, Institut de Biologie de l'École Normale Supérieure (IBENS), France

Muhammed Simsek, McMaster University, Canada

Esteban Finol, National University of Singapore, Singapore

Megan Frederickson, University of Toronto, Canada

Sudarshan Gadhadhar, Institute for Stem Cell Science and Regenerative Medicine, India

Gina Galli, University of Manchester, UK

Nick Gatford, University of Oxford, UK

Mark Gibson, Escola Universitária Vasco da Gama, Portugal

Benjamin Glass, University of Pennsylvania, USA

Susan Glendinning, University of the Sunshine Coast, Australia

Emily Glover, University of Bristol, UK

Daniela Goffré, National University of Quilmes, Argentina

Caglar Gok, University of Lincoln, UK

Alisson Gontijo, University of Lisbon, Portugal

Nicanor González-Morales, Dalhousie University, Canada

Sara Goodwin, Cold Spring Harbor Laboratory, USA

Etika Goyal, Marine Biological Laboratory, USA

Xavier Grau-Bové, Centre for Genomic Regulation, Spain

Fiorito Graziano, Stazione Zoologica Anton Dohrn Napoli, Italy

David Green, Campbell University, USA

Kendra Greenlee, North Dakota State University, USA

Yaodong Gu, University of Auckland, New Zealand

Peter Gunning, University of New South Wales, Australia

Fouzia Haider, Carleton University, Canada

Mark Hanson, University of Exeter, UK

Joshua Hare, University of Miami Miller School of Medicine, USA

Gaelle Hayot, Karlsruhe Institute of Technology, Germany

Jinwu He, Kunming Institute of Zoology, China

Greg Hermann, Lewis & Clark College, USA

Patricia Hernandez, Columbian College of Arts and Sciences, USA

Nathaniel Himmel, University of Lausanne, Switzerland

Kristen Hogan, University of South Carolina, USA

Vishnu Hosur, The Jackson Laboratory, USA

Robert Hynds, University College London, UK

Juan Ianowski, University of Saskatchewan, Canada

Igor Iatsenko, Max Planck Institute for Infection Biology, Germany

Cathy Jackson, Institut Jacques Monod, France

Dan Jagger, University College London Ear Institute, UK

Krzysztof Jagla, University of Clermont Auvergne, France

Sonal Jaiswal, Eberhard Karls University of Tübingen, Germany

T. C. Jermin Jeaunita, Loyola Institute of Technology and Science, India

Neil Kad, University of Kent, UK

Alex Keene, Texas A&M University, USA

Markku Keinanen, University of Eastern Finland, Finland

Allison Kennedy, Sanofi Pasteur, Canada

Justin Kenney, Wayne State University, USA

Biology Open • Supplementary information

Hemant Khanna, Astellas Pharma Inc, USA

Shaun Killen, University of Glasgow, UK

Roger Kissane, University of Liverpool, UK

Kalyan Kondapalli, University of Michigan-Dearborn, USA

Sandhya Koushika, Tata Institute of Fundamental Research, India

Patricia Kramer, University of Washington, USA

Mira Krendel, SUNY Upstate Medical University, USA

Yung-Shu Kuan, National Taiwan University, Taiwan

Adam Kwiatkowski, University of Pittsburgh, USA

Raj Ladher, National Centre for Biological Sciences, India

Izzy Langley, University of St Andrews Sea Mammal Research Unit, UK

Kaitlin Laws, Randolph Macon College, USA

Chris Lawson, University of Queensland, Australia

Youngsoo Lee, Ajou University School of Medicine, Republic of Korea

Adele Leggieri, University College London, UK

Felix Leiva, Radboud University, Netherlands

Muheng Liao, The University of Hong Kong, Hong Kong

Jeremy Logue, Albany Medical College, USA

Holly Lovegrove, University of Manchester, UK

Fayaz A. Malik, Council of Scientific and Industrial Research – Indian Institute of Integrative Medicine (CSIR-IIIM), India

Aarren Mannion, Max Planck Institute for Molecular Biomedicine, Germany

Miguel Manzanares, Severo Ochoa Centre for Molecular Biology, Spain

Ruben Marin-Juez, Sainte-Justine University Hospital Research Center, Canada

Benjamin Marshall, University of Glasgow, UK

Arnaud Martin, George Washington University, USA

J.P. Martinez-Barbera, University College London, UK

L. Miguel Martins, MRC Toxicology Unit, UK

Elvira Mass, University of Bonn, Germany

Jennifer Mather, University of Lethbridge, Canada

Kenneth McElreavey, Pasteur Institute, France

Christopher McGinnis, Stanford University, USA

Daniel McIntyre, Duke University, USA

Luis Menezes, National Institutes of Health, USA

Julian Mensch, University of Buenos Aires, Argentina

Marco Milan, Institute for Research in Biomedicine (IRB Barcelona), Spain

Anzy Miller, University of Manchester, UK

Adaobi Mofunanya, Farmingdale State College, USA

Hernan Morales-Navarrete, Universidad de Las Americas, Ecuador

Paulina Moreno-Layseca, University of Turku, Finland

Patrick Narbonne, University of Quebec, Canada

Qamariya Nasrullah, King's College London, UK

Bhagwat Nawade, Western Sydney University, Australia

Francesca Nazio, University of Rome Tor Vergata, Italy

Vidya Negi, Indian Institute of Science Education and Research, India

James Nieh, University of California, San Diego, USA

Goran Nilsson, University of Oslo, Norway

Tommy Norin, Technical University of Denmark, Denmark

Jordana Oliveira, University of Ottawa, Canada

Marcus F. Oliveira, Institute of Medical Biochemistry (IBqM), Brazil

Thomas O'Mahoney, Anglia Ruskin University, UK

Julie Ostrander, University of Minnesota Medical School, USA

Indira Paddibhatla, Johns Hopkins University School of Medicine, USA

Alexandre Paix, Max Planck Institute for Biology, Germany

Toni R. Pak, Loyola University Chicago, USA

Giuseppe Palaia, Polytechnic of Turin, Italy

Matthew Pamenter, University of Ottawa, Canada

Udai Pandey, University of Pittsburgh Medical Center, USA

Matthew Parker, University of Surrey, UK

Barbara Pernaute, Andalusian Center for Developmental Biology (CABD), Spain

Marine Petit, University of Surrey, UK

Patrice Petit, Saints-Pères Paris Institute for the Neurosciences, France

Berenika Plusa, University of Manchester, UK

Sreenivasan Ponnambalam, University of Leeds, UK

Andrea Pozzi, Monash University, Australia

Hamed Rajabi, London South Bank University, UK

Navin Ramakrishna, Genome Institute of Singapore, Singapore

Soundhar Ramasamy, Weill Cornell Medicine, USA

Ben Raymond, University of Exeter, UK

Alessio Reggio, Saint Camillus International University of Health and Medical Sciences, Italy

Joanna Reinhold, Longwood University, USA

Arun Renganathan, Washington University School of Medicine in Saint Louis, USA

Joerg Renkawitz, Ludwig Maximilians University Munich, Germany

Samuel Robinson, Institute for Wetland and Waterfowl Research, Canada

Aida Rodrigo Albors, University of Edinburgh, UK

Roberto Rodriguez, University of Puerto Rico, USA

Javier Rodríguez-Baena, Institute for Neurosciences CSIC-UMH, Spain

Michael Romero, Mayo Clinic College of Medicine and Science, USA

Julien Royet, Aix-Marseille University, France

Dimitri Ryczko, University of Sherbrooke, Canada

Madhumala Sadanandappa, Dartmouth Hitchcock Medical Center, USA

Hugo Sanchez-Castillo, National Autonomous University of Mexico, Mexico

Estefania Sanchez-Vasquez, University of California, Santa Cruz, USA

Kirankumar Santhakumar, SRM Institute of Science and Technology, India

Pierre Santucci, Aix-Marseille University, France

Noriaki Sasai, Nara Institute of Science and Technology, Japan

Stefan Schulte-Merker, University of Münster, Germany

Camila Scorticati, Universidad Nacional de San Martin, Argentina

Lindsey Seldin, Emory University, USA

Bhagyashree Senapati, University of Oxford, UK

Ryan Sheldon, Van Andel Institute, USA

Tal Shomrat, Ruppin Academic Center, Israel

Ankit Shroff, University of Nebraska Medical Center, USA

Glenn Simmons, Cornell University, USA

Filipa Simões, University of Oxford, UK

Sumeet Singh, Université Libre de Bruxelles, Belgium

Victoria Spencer, Gregor Mendel Institute of Molecular Plant Biology, Germany

Claudia Spits, Université Libre de Bruxelles, Belgium

Marcus Stensmyr, Lund University, Sweden

Nicola Stevenson, University of Bristol, UK

Marina Stojanova, Saints Cyril and Methodius University in Skopje, North Macedonia

Rajivgandhi Sundaram, Johns Hopkins University School of Medicine, USA

Gregory P. Sutton, University of Lincoln, UK

Sheyum Syed, University of Miami, USA

Mohita Tagore, Memorial Sloan Kettering Cancer Center, USA

David R. Tarpy, North Carolina State University, USA

Eli Thoré, University of Namur, Belgium

Dhanendra Tomar, Wake Forest University School of Medicine, USA

Drenka Trivanovic, University of Belgrade, Serbia

Takuya Uehara, National Agriculture and Food Research Organization, Japan

Václav Uruba, University of West Bohemia, Czech Republic

Erik van Bergen, University of Lisbon, Portugal

Freek van Eeden, University of Sheffield, UK

Saranyaraajan Varadarajan, University of Michigan Medicine, USA

Katrina Velle, University of Massachusetts Amherst, USA

Wilco Verberk, Radboud University, Netherlands

Julian Vincent, Heriot-Watt University, UK

Alessandra Vitaliti, University of Rome Tor Vergata, Italy

Miloš Vittori, University of Ljubljana, Slovenia

Anastasios Vourekas, Louisiana State University, USA

Shuoshuo Wang, Beth Israel Deaconess Medical Center, USA

Tobias Wang, Aarhus University, Denmark

Lukas Weiss, Princeton University, USA

Brooke Whitelaw, University of Otago, New Zealand

Alaina Willet, Vanderbilt University, USA

Cassondra Williams, National Marine Mammal Foundation, USA

Jessie Williamson, University of Wyoming, USA

Sara M. Wilmsen, University of Virginia, USA

Jia-Ping Wu, Shaoguan University, China

Nicholas Wu, Murdoch University, Australia

Kang Nian Yap, Norwegian University of Science and Technology, Norway

Bing Ye, University of Michigan, USA

Min Zhang, Capital Medical University, China

Xincheng Zhao, Henan Agricultural University, China

Chaogu Zheng, The University of Hong Kong, Hong Kong
